# Supplementary material for: Genotype‒phenotype correlation in recessive DNAJB4 myopathy
Source: Acta Neuropathol Commun. 2024 Oct 28;12:171. doi: 10.1186/s40478-024-01878-w (PMC11514740; doi:10.1186/s40478-024-01878-w)
Supplement: Supplementary file 1 — Supplementary Material 1 [file 40478_2024_1878_MOESM1_ESM.docx]

**SUPPLEMENTARY MATERIALS**

**Methods**

Genetic testing

For Family 1(BOS_0868), Illumina-based short read whole exome sequencing was performed on genomic DNA extracted from whole blood by the Genomics Platform of the Broad Institute of MIT and Harvard as described [1]. Variants were filtered and annotated on the seqr platform [2].

For Family 2 (BOS_1575), variants in FASTQ files from previous clinical whole exome sequencing by GeneDx was re-called and analyzed on the variant Explorer Pipeline (VExP), as described [3], and underwent research-based reanalysis by the Manton Center Gene Discovery Core.

For Family 3, genomic DNA was isolated from peripheral blood and the exome sequencing libraries were prepared with QIAseq Human Exome Kit (Qiagen) according to the manufacturer's instructions. After quality control, the sample were sequenced on Illumina NovaSeq 6000 system with average sequencing depth of ×122. The FASTQ files generated from sequencing were uploaded to the Genomize Seq software v8.4.0 (Genomize, Turkey). Subsequently, the reads were aligned to the human reference genome GRCh37 (hg19) using the Burrows-Wheeler Aligner, and variant calling and annotation were performed with the Freebayes and Variant Effect Predictor tools, respectively. Three years after the initial study, in 2023, the causal variant was identified by reanalysis of the exome data with the literature [4].

For Family 4, exome sequencing was performed by Mendelics Genomic Analysis (São Paulo, SP, Brazil) as previously described [5]. Target regions capture through probes. Next generation sequencing using Illumina technology. Alignment and variant calling were performed through bioinformatic pipelines using the human genome version GRCh38 as reference. The aligned BAM files were processed by the ExomeDepth software, an R package destined to identify copy number variations (CNV). Medical analysis guided by clinical referring information. Use of GnomAD for variant allele frequency reference.

For Family 5, whole-exome sequencing analysis was performed as described [6].

**References**

1. Wojcik MH, Thiele K, Grant CF, Chao K, Goodrich J, O’Donnell-Luria A, et al. Genome Sequencing Identifies the Pathogenic Variant Missed by Prior Testing in an Infant with Marfan Syndrome. *J Pediatr* 2019;213:235–40. https://doi.org/10.1016/j.jpeds.2019.05.029

2 Pais LS, Snow H, Weisburd B, Zhang S, Baxter SM, DiTroia S, et al. seqr: A web‐based analysis and collaboration tool for rare disease genomics. Hum Mutat 2022;43:698–707. https://doi.org/10.1002/humu.24366

3. Schmitz-Abe K, Li Q, Rosen SM, Nori N, Madden JA, Genetti CA, et al. Unique bioinformatic approach and comprehensive reanalysis improve diagnostic yield of clinical exomes. *Eur J Hum Genet* 2019;27:1398–405. https://doi.org/10.1038/s41431-019-0401-x

4. Weihl CC, Töpf A, Bengoechea R, Duff J, Charlton R, Garcia SK, et al. Loss of function variants in DNAJB4 cause a myopathy with early respiratory failure. *Acta Neuropathol*. 2023;145:127–43. https://doi.org/10.1007/s00401-022-02510-8

5. Barcelos IP de, Bueno C, Godoy LFS, Pessoa A, Costa LA, Monti FC, et al. Subacute Partially Reversible Leukoencephalopathy Expands the Aicardi–Goutières Syndrome Phenotype. *Brain Sci* 2023;13:1169. https://doi.org/10.3390/brainsci13081169

6. Al-Kasbi G, Al-Murshedi F, Al-Kindi A, Al-Hashimi N, Al-Thihli K, Al-Saegh A, et al. The diagnostic yield, candidate genes, and pitfalls for a genetic study of intellectual disability in 118 middle eastern families. *Sci Rep* 2022;12:18862. https://doi.org/10.1038/s41598-022-22036-z
